# A long-term field experiment demonstrates the influence of tillage on the bacterial potential to produce soil structure-stabilizing agents such as exopolysaccharides and lipopolysaccharides

**DOI:** 10.1186/s40793-019-0341-7

**Published:** 2019-03-28

**Authors:** Barbara Cania, Gisle Vestergaard, Maike Krauss, Andreas Fliessbach, Michael Schloter, Stefanie Schulz

**Affiliations:** 1 Research Unit Comparative Microbiome Analysis, Helmholtz Zentrum München Deutsches Forschungszentrum für Gesundheit und Umwelt (GmbH), Ingolstädter Landstraße 1, 85764 Neuherberg, Germany; 2 Department of Soil Sciences, Research Institute of Organic Agriculture (FiBL), Ackerstrasse 113, 5070 Frick, Switzerland; 3 Soil Science, Technical University of Munich, Emil-Ramann-Straße 2, 85354 Freising, Germany; 4 Section for Bioinformatics, Department of Health Technology, Technical University of Denmark, 2800 Lyngby, Denmark

**Keywords:** Tillage, Soil aggregates, Exopolysaccharides, Lipopolysaccharides, Soil microbiome, Metagenomics, *wza*, *lptF*, *lptG*

## Abstract

**Background:**

Stable soil aggregates are essential for optimal crop growth and preventing soil erosion. However, tillage is often used in agriculture to loosen the soil, which disrupts the integrity of these aggregates. Soil aggregation can be enhanced by bacteria through their ability to produce exopolysaccharides and lipopolysaccharides. These compounds stabilize soil aggregates by “gluing” soil particles together. However, it has yet to be shown how tillage influences the bacterial potential to produce aggregate-stabilizing agents. Therefore, we sampled conventional and reduced tillage treatments at 0–10 cm, 10–20 cm and 20–50 cm from a long-term field trial in Frick, Switzerland. We compared the stable aggregate fraction of the soil and the bacterial potential to produce exopolysaccharides (EPS) and lipopolysaccharides (LPS) under different tillage regimes by employing a shotgun metagenomic approach. We established a method which combines hidden Markov model searches with blasts against sequences derived from the Kyoto Encyclopedia of Genes and Genomes database to analyze genes specific for the biosynthesis of these compounds.

**Results:**

Our data revealed that the stable aggregate fraction as well as the bacterial potential to produce EPS and LPS were comparable under both tillage regimes. The highest potential to produce these compounds was found in the upper soil layer, which was disturbed by tillage, but had higher content of organic carbon compared to the layer below the tillage horizon. Additionally, key players of EPS and LPS production differed at different sampling depths. Some families with high potential to produce EPS and LPS, such as *Chitinophagaceae* and *Bradyrhizobiaceae*, were more abundant in the upper soil layers, while others, e.g. *Nitrospiraceae* and *Planctomycetaceae*, preferred the lowest sampled soil depth. Each family had the potential to form a limited number of different aggregate-stabilizing agents.

**Conclusions:**

Our results indicate that conventional tillage and reduced tillage equally promote the bacterial potential to produce EPS and LPS in the tillage horizon. However, as major bacterial groups triggering EPS and LPS formation were not the same, it is likely that gene expression pattern differ in the different treatments due to various pathways of gene induction and transcription in different bacterial species.

**Electronic supplementary material:**

The online version of this article (10.1186/s40793-019-0341-7) contains supplementary material, which is available to authorized users.

## Background

Globally, 33% of land resources have been classified as moderately to highly degraded [1]. The main causes of soil degradation are poor agricultural management practices, such as conventional tillage (CT), which lead to erosion, loss of soil organic carbon and nutrient imbalance [2]. It turns out that a combination of reduced tillage (RT) and organic farming (OF) is a good compromise to diminish the aforementioned problems [3–5]. However, RT is still not commonly used by organic farmers due to increased weed pressure, topsoil compaction and restricted N availability, which may compromise yield [6, 7]. As even one-time ploughing may counteract the benefits of RT, these practices need to be developed further under long-term OF [8, 9].

One of the advantages of RT over CT practices is the better preservation of soil aggregates [10, 11]. The presence of stable aggregates defines good soil structure, which improves crop growth and prevents erosion [12, 13]. The stability of aggregates strongly depends on their size. Microaggregates (< 250 μm) form slower than macroaggregates (> 250 μm), but they are also more stable, even under unfavorable soil management systems [14, 15]. Aggregate formation results from complex interactions between soil fauna, microorganisms, roots, inorganic binding agents and different environmental variables. Fungi have been considered as the most important microorganisms involved in the formation of macroaggregates due to their hyphal structure [15, 16]. In contrast, bacteria are of higher importance for soil aggregation at the microscale, as they are capable of synthesizing exopolysaccharides (EPS) and lipopolysaccharides (LPS), which act as “glue” for soil particles [15, 16]. Bacteria use these compounds for cell attachment to mineral surfaces, which fosters the formation of composite building units and microaggregates [15–17]. While EPS are a very diverse group of high-molecular-weight polymers composed of sugar residues, LPS share a common structure. The number of possible EPS structures is almost infinite [18]. Most EPS are initially synthesized intracellularly and then secreted to the external environment, which requires the contribution of at least three gene families: I) genes encoding for enzymes involved in biosynthesis of nucleotide sugars, II) genes encoding for glycosyltransferases, which catalyze transfer of the nucleotide sugars from activated donor molecules to specific acceptors in the plasma membrane, and III) genes encoding for proteins involved in EPS assembly and export [19]. Alternatively, EPS can be synthesized extracellularly by different synthase proteins [20]. Most enzymes involved in the EPS biosynthesis are strain-specific and can catalyze multiple metabolic processes.

LPS are glycolipids that are comprised of a lipid moiety (lipid A) and a polysaccharide (composed of O-antigen, outer core and inner core), both with variable structures [21]. These parts are synthesized independently inside a cell, and then ligated together at the inner membrane, forming a mature LPS. The mature molecule is transported to the cell surface by several proteins that form an LPS export complex. As in EPS biosynthesis, very few of these proteins are conserved and catalyze LPS production only [22].

The gluing properties of both types of polysaccharides could be crucial in agricultural soils, as it was demonstrated that even slight changes in the sugar composition drastically changed the physical properties of the polysaccharide [18]. Consequently, tillage might not only change the bacterial community composition in soil [23], but also the composition of EPS/LPS, and thus affect aggregate stability and de novo formation after disturbance.

The synthesis of EPS and LPS requires both high levels of energy and easily accessible carbon. Especially under CT, reduced soil organic carbon stocks have been frequently observed [4, 23, 24]. Therefore, we hypothesized that under long-term CT, abundance of EPS and LPS forming bacteria would be reduced compared to RT. To investigate this, high-throughput shotgun sequencing was used to obtain metagenomic information on microbiomes of three soil layers (0–10 cm, 10–20 cm and 20–50 cm) under RT and CT management from a long-term organic field trial in Frick (Switzerland). To analyze genes specific for EPS and LPS production, we used an approach which combined hidden Markov model (HMM) searches with blasts against sequences derived from the Kyoto Encyclopedia of Genes and Genomes (KEGG) database. As we investigated bacterial potentials samples were taken in spring where an influence of plants and fertilization could be excluded.

## Materials and methods

### Site description and soil sampling

Soil samples were taken from a long-term trial in Frick, Switzerland (47°30′N, 8°01′E, 350 m a.s.l.), established in 2002 by the Research Institute of Organic Agriculture (FiBL). The site was under conventional management until 1995, when it changed to organic standards in accordance with the European Union Regulation (EEC) No. 2092/91. The mean annual precipitation and temperature are 1000 mm and 8.9 °C, respectively. The soil is a Stagnic Eutric Cambisol with a pH of 7.1 and composed of 22% sand, 33% silt and 45% clay. The factorial design includes the factors tillage, fertilisation and biodynamic preparations and has been described in detail by Berner et al. [3].

In this study, only the two tillage treatments were compared: conventional tillage (CT) with a mouldboard plough operating at 15–18 cm depth, and reduced tillage (RT) with a chisel and a skim plough (5–10 cm) used to loosen the soil. In both systems, seedbed preparation was performed with a rotary harrow running at a depth of 5 cm. The usage of standard farming equipment was made possible by the plot size (12 m × 12 m). The plots were arranged in a strip-split-plot design.

Samples were taken from three out of four replicated plots per tillage system in the slurry fertilized plots without biodynamic preparations in March 2015 in a green manure ley, before tillage and subsequent maize cropping started. In 2014, winter wheat was harvested in July, followed by the seeding of a green manure mixture (Orgamix DS, *Trifolium incarnatum, Vicia villosa, Avena sativa*) in August, which was harvested in April 2015. All plots were fertilized with slurry during the wheat growing season in 2014 (the exact dates and fertilization details are summarized in Additional file 1). Soil samples were taken using a soil auger with a diameter of 2.5 cm. Approximately 10 cores per plot were sampled to a soil depth of 50 cm. Each soil core was divided into three layers: 0–10 cm, 10–20 cm and 20–50 cm. Samples from the same layer of each plot were homogenized, resulting in 18 samples (3 depths × 2 tillage treatments × 3 plot replicates). The samples were directly cooled in the field and either processed immediately (biochemical analyses) or stored in − 20 °C until processing (DNA extraction and sequencing).

### Physical, chemical and major biological properties of soils

We determined the stable aggregate fraction (SAF) of the soils by a wet sieving technique, where 5 g of moist soil was immersed in water using a sieving apparatus according to Murer et al. [25]. After 5 min of moving the sieves up and down in the water phase, the remainder on the sieve consisting of aggregates and particles > 0.25 mm was dried at 105 °C. The aggregates were then destroyed by adding a 0.1 M Na_4_P_2_O_7_ solution, leaving only particles > 0.25 mm (sand and organic debris) on the sieve that were dried again. Apart from using moist soil without further fractionation, the method follows the details as given by Murer et al. [25].

Soil organic carbon (SOC) concentration was determined by wet oxidation of 1 g of air-dried and ground soil in 20 ml of concentrated H_2_SO_4_ and 25 ml of 2 M K_2_Cr_2_O_7_. The determination of dissolved organic carbon (DOC) and microbial biomass carbon (Cmic) was accomplished by means of a chloroform fumigation extraction method (CFE) using 20 g of moist soil, sieved on a 5 mm sieve. 0.5 M K_2_SO_4_ solution was added at a weight to volume (*w*/*v*) ratio of 1:4. Subsequently, measurements were performed using a TOC/TNb analyzer (Analytik Jena AG, Germany). DOC was determined from the non-fumigated samples, and Cmic was calculated as a difference between the fumigated and the non-fumigated samples. The assessment of SOC and Cmic was described in detail by Krauss et al. [23].

### DNA extraction and sequencing

Total nucleic acids were directly extracted from 0.5 g of frozen soil according to the phenol-chloroform based DNA/RNA coextraction protocol described by Lueders et al. [26]. Beat beating was performed by means of CKMix tubes and a Precellys24 homogenizer (Bertin Technologies, France). Extracted DNA was checked for purity using a NanoDrop 1000 spectrophotometer (Thermo Fisher Scientific, USA). The quantity was also verified by means of a Quant-iT PicoGreen dsDNA Assay Kit (Life Technologies, USA). Extracted DNA was then stored in − 20 °C until further processing.

One microgram of DNA from each sample was sheared using an E220 Focused-ultrasonicator (Covaris, USA), following the manufacturer’s guideline for the target size of 500 bp (conditions: peak incident power – 175 W, duty factor – 5%, cycles per burst – 200, treatment time – 35 s, temperature – 7 °C, water level – 6, sample volume – 50 μl, intensifier – yes). Libraries were prepared with 50–100 ng of the sheared DNA, using a NEBNext Ultra DNA Library Prep Kit for Illumina, and NEBNext Multiplex Oligos for Illumina (New England Biolabs, UK) as barcodes. According to the manufacturer’s manual, the NEBNext Adaptor from Illumina was diluted 10-fold to prevent the occurrence of dimers. Size selection was performed with Agencourt AMPure XP beads (Beckman Coulter, USA), using the volumes selecting for libraries with 400–500 bp inserts. The AMPure XP beads were also used for cleanup of PCR amplification and a following additional cleanup step to eliminate the residual primer dimers (1:0.6 DNA to bead ratio).

Library size was estimated using High Sensitivity DNA Analysis Kits together with a 2100 Bioanalyzer (Agilent, USA). DNA concentration was subsequently assessed by means of a Quant-iT PicoGreen dsDNA Assay Kit. Libraries were then diluted to a concentration of 4 nM each and pooled equimolar. 10 pM of the mixture was spiked with 30% PhiX, used as a quality and calibration control [27], and sequenced on a MiSeq sequencer using a MiSeq Reagent Kit v3 for 600 cycle (Illumina, USA).

### Data filtering and taxonomic analysis

Raw sequencing data attained from the MiSeq was filtered according to Vestergaard et al. [28] by removing remnant adaptor sequences and trimming the reads. This was accomplished by using AdapterRemoval [29] set to: 5′/3′ terminal minimum Phred quality = 15, minimum read length = 50. PhiX contamination was removed using DeconSeq [30]. For taxonomic annotation, filtered reads were blasted against the National Center for Biotechnology Information Non-Redundant (NCBI-NR) protein sequences database (October 2015) using Diamond (version 0.5.2.32) with sensitive parameters [31]. Based on the top 25 blast results (i.e. hits with the lowest e-value), a unique taxon ID was assigned to each filtered read with the MEtaGenome Analyzer software (MEGAN, version 5.10.6) [32]. During the MEGAN analysis, the following parameters were applied: MinScore = 50.0, MaxExpected = 0.01, TopPercent = 10.0, MinSupport = 1, MinComplexity = 0. Additionally, 16S rRNA gene sequences were identified using SortMeRNA (version 2.0) [33]. Taxonomy was assigned to those reads using QIIME (version 1.9.1) [34] based on the SILVA database (release 123).

### Functional analysis

Protein sequences associated with EPS and LPS biosynthesis and excretion were downloaded directly from the online Kyoto Encyclopedia of Genes and Genomes (KEGG) Orthology database (October 2016). They were examined for the presence of function-specific conserved domains using CD-search [35]. KEGG Orthology (KO) entries which contained such domains were then used to construct specific databases by means of Diamond. Hidden Markov models (HMMs) of corresponding conserved domains were obtained from the TIGRFAMs database (version 15) [36] and the Pfam database (version 30) [37]. FragGeneScan (version 1.19) [38] was used on the filtered sequencing reads to predict open-reading frames, which were subsequently scanned with HMMER (version 3) (hmmer.org). Reads matching the downloaded HMMs (E-value threshold = 10^− 5^), were blasted against the self-built KO databases. A KO ID was assigned to those reads for which the top 25 blast results were consistent. The specificity of this approach was verified by using blastx against the Non-redundant protein sequences (nr) database. Out of 81 examined KO numbers (67 for EPS and 14 for LPS), 14 gave sufficiently specific results. The results were considered sufficiently specific if 25 randomly selected reads (or all if less reads were assigned) per a KO number were assigned to the function of interest. Analysis of EPS and LPS biosynthesis and excretion was performed using separate databases. Open-reading frames of the assigned reads were searched against the full Pfam and TIGRFAMs databases. This resulted in 81.8% of the reads matching the downloaded HMMs. All examined KO numbers are listed in Additional file 2 and the HMMs and KO numbers used for the analysis are summarized in Table 1.

**Table 1:** Proteins related to exo- and lipopolysaccharide production with corresponding KO numbers, HMM IDs and genes

Protein	KO number	HMM ID	Gene
polysaccharide export outer membrane protein Wza	K01991	PF02563	*wza*
alginate export outer membrane protein AlgE	K16081	PF13372	*algE*
alginate biosynthesis acetyltransferase AlgJ	K19295	PF16822	*algJ*
colanic acid biosynthesis acetyltransferase WcaB	K03819	TIGR04016	*wcaB*
colanic acid biosynthesis acetyltransferase WcaF	K03818	TIGR04008	*wcaF*
colanic acid/amylovoran biosynthesis pyruvyl transferase WcaK/AmsJ	K16710	TIGR04006	*wcaK*/*amsJ*
capsular polysaccharide export system permease KpsE	K10107	TIGR01010	*kpsE*
exopolysaccharide biosynthesis transmembrane protein EpsG	K19419	PF14897	*epsG*
exopolysaccharide biosynthesis tyrosine kinase modulator EpsA	K19420	TIGR01006	*epsA*
levansucrase SacB	K00692	PF02435	*sacB*
lipopolysaccharide transport system ATP-binding protein Wzt	K09691	PF14524	*wzt*
LptBFGC lipopolysaccharide export complex permease LptF	K07091	TIGR04407	*lptF*
LptBFGC lipopolysaccharide export complex permease LptG	K11720	TIGR04408, PF03739	*lptG*
LptBFGC lipopolysaccharide export complex inner membrane protein LptC	K11719	TIGR04409, PF06835	*lptC*

### Statistical analysis and data visualization

All statistical analyses were conducted using R version 3 [39]. Metagenomic datasets were analyzed based on relative abundances of reads. These were obtained by dividing the number of reads assigned to a gene or organism by the total number of filtered reads per sample, and multiplying by 100. Effects of tillage, depth and their possible interaction were detected by multilevel models. For this purpose, the lme function from the nlme package was used [40]. The influence was considered significant when the *p*-value was below 5% (*P* < 0.05). Differences between sampled depths were identified by setting the following contrasts: 0–20 cm vs 20–50 cm and 0–10 cm vs 10–20 cm. For data derived from the metagenomic datasets, the Benjamini-Hochberg procedure was performed prior to analyzing contrasts. The Shannon-Wiener index was calculated using the alpha.div function of the R asbio package to measure diversity within the samples [41]. To visualize the level of dissimilarity between the samples, non-metric multidimensional scaling (NMDS) ordination plots were created based on the Bray-Curtis distance metrics, using the metaMDS function in the R vegan package [42]. The core microbiomes were identified by means of InteractiVenn [43]. For the purpose of calculating these cores, a family was recognized as present in a treatment only if it was detected in at least two out of three replicates.

## Results

### Soil properties

The stable aggregate fraction (SAF) of the soil, soil organic carbon (SOC), dissolved organic carbon (DOC) and microbial biomass carbon (Cmic) data is summarized in Table 2. Aggregate stability was highest in the 20–50 cm depth, and did not differ significantly between the upper depths. It was also not significantly influenced by tillage. SOC stocks decreased with depth, and were higher in the 0–20 cm depth under RT compared to CT. DOC concentrations were highest in the 0–10 cm depth under RT, and showed little difference between the other samples. Microbial biomass decreased with depth, and was more stratified under RT. In the 0–10 cm depth, Cmic values were higher under RT.

**Table 2:** Stable aggregate fraction of the soil, carbon stocks and microbial biomass

Tillage	Conventional			Reduced		
Depth	0–10 cm	10–20 cm	20–50 cm	0–10 cm	10–20 cm	20–50 cm
SAF (%)^*^	56.19 ± 8.98	48.58 ± 3.05	65.21 ± 9.62	50.35 ± 8.30	51.52 ± 6.73	69.02 ± 2.26
SOC (%)^#^	2.30 ± 0.41	2.15 ± 0.33	1.25 ± 0.37	2.92 ± 0.28	2.31 ± 0.26	1.23 ± 0.22
DOC (mg kg^− 1^)^#^	62.44 ± 9.79	53.61 ± 15.84	58.41 ± 10.23	99.49 ± 19.84	62.32 ± 7.62	51.97 ± 8.45
Cmic (mg kg^−1^)^#^	981.81 ± 158.92	849.18 ± 106.23	352.12 ± 121.46	1306.73 ± 122.07	932.26 ± 67.23	374.75 ± 58.22

*SAF* Stable aggregate fraction, *SOC* Soil organic carbon, *DOC* Dissolved organic carbon, and *Cmic* microbial biomass carbon values of soils under two tillage systems, sampled at three different depths. Average values and standard deviations (±) are calculated based on triplicates (*n* = 3). Detected influence (*p* < 0.05) is symbolized for depth (*) or interaction of tillage and depth (#), respectively

### Shotgun sequencing characteristics

Shotgun sequencing of the 18 libraries, prepared from two tillage treatments – conventional (CT) and reduced (RT) – sampled at three depths (0–10 cm, 10–20 cm and 20–50 cm) from three independent plots treated as replicates, generated 11.8 gigabases of data in total. This corresponded to 39.307.875 filtered reads with an average length after trimming of 297 bp. Details of the sequencing run are summarized in Additional file 3.

### Taxonomic analysis of the general bacterial community

When all filtered reads were blasted against the NCBI-NR database, 55.8% were assigned to Bacteria, 1.2% to Archaea, 1.3% to Fungi and 41.7% to others. Further analysis focused on bacteria and was conducted at the level of family, at which 21.1% of filtered reads were assigned. In total, bacteria comprised 296 families.

The non-metric multidimensional scaling (NMDS) ordination plot (Additional file 4A) showed a difference between the composition of bacterial families originating from the deepest sampled soil layer (20–50 cm) and the upper soil layers (0–10 cm and 10–20 cm), but revealed no clear separation of the tillage treatments. This was confirmed by means of a multilevel model. Abundances of 103 families were influenced by depth, while none was affected by tillage, and one by the interaction of both factors. The full list of impacted families can be taken from Additional file 5.

**Fig. 1:**
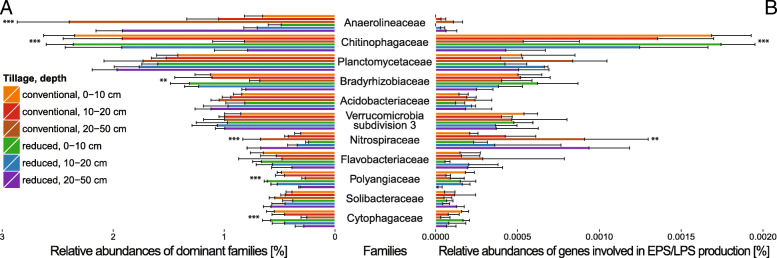
Dominant families and their potential to produce EPS and LPS. Comparison of (A) abundances of dominant bacterial families whose relative abundances exceeded 0.5%, with (B) their potential to produce EPS and LPS. Taxonomic assignment was performed against the National Center for Biotechnology Information Non-Redundant (NCBI-NR) protein sequences database. Functional genes were assigned using hidden Markov models (HMMs) obtained from the TIGRFAMs and Pfam databases, and then sequences derived from the Kyoto Encyclopedia of Genes and Genomes (KEGG) Orthology database. Significant differences in the amount of annotated reads were determined by a multilevel model (n = 3). Detected influence is symbolized for depth (*) or interaction of tillage and depth (#), respectively. Significance levels are represented by the amount of symbols: 1 – p < 0.05, 2 – *p* < 0.01, 3 – *p* < 0.001. Error bars show standard deviation

In-depth analysis of the effects of tillage, depth and their interaction on the general community structure was performed on dominant families whose abundance exceeded 0.5% (Fig. 1a). The most abundant family, *Anaerolineaceae*, together with *Nitrospiraceae*, were found mainly in 20–50 cm. *Chitinophagaceae*, *Bradyrhizobiaceae*, *Polyangiaceae* and *Cytophagaceae* had higher abundance in 0–20 cm. *Planctomycetaceae*, *Acidobacteriaceae*, *Verrucomicrobia subdivision 3*, *Flavobacteriaceae* and *Solibacteraceae* were not significantly influenced by either depth or tillage.

The results from the taxonomic analysis encompassing the entire metagenomic datasets were supported by SILVA’s taxonomic annotations of the 16S rRNA gene. Of all filtered reads, 0.21% were assigned to the 16S rRNA gene. With both approaches, the bacterial communities showed similar distribution patterns and one third of the dominant families remained the same, with *Anaerolineaceae* staying the most abundant, regardless of the assignment method used (Additional file 6).

### Relative abundances of genes catalyzing EPS and LPS synthesis and excretion

An approach combining hidden Markov model (HMM) searches with blasts against sequences derived from the Kyoto Encyclopedia of Genes and Genomes (KEGG) database was used to target genes specific for the biosynthesis and excretion of alginate, colanic acid, levan and other EPS, as well as LPS (Table 1). Sufficient coverage of the diversity of the analyzed genes was confirmed by performing explanatory rarefaction analysis (Additional file 7).

**Fig. 2:**
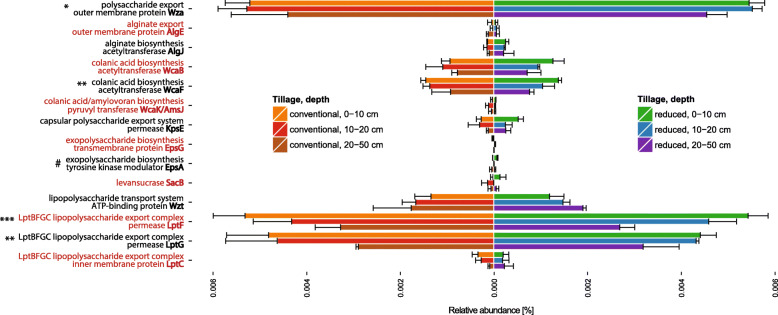
Genes encoding for proteins involved in biosynthesis of EPS and LPS. Relative abundances of genes encoding for proteins involved in biosynthesis of EPS and LPS in soils under two tillage managements, sampled at three different depths. Functional genes were assigned using hidden Markov models (HMMs) obtained from the TIGRFAMs and Pfam databases, and then sequences derived from the Kyoto Encyclopedia of Genes and Genomes (KEGG) Orthology database. Significant differences in the amount of annotated reads were determined by a multilevel model (n = 3). Detected influence is symbolized for depth (*) or interaction of tillage and depth (#), respectively. Significance levels are represented by the amount of symbols: 1 – p < 0.05, 2 – p < 0.01, 3 – p < 0.001. Error bars show standard deviation

In total, the investigated genes comprised 0.018% of all filtered reads (Fig. 2). Dominant genes, with a relative abundance above 0.005% of total reads, were *wza*, *lptF* and *lptG*, which encode for an outer membrane protein responsible for EPS excretion, and permeases of the LptBFGC LPS export system, respectively. Moderately abundant genes (> 0.001%) were *wcaB*, wcaF (encoding for a colanic acid biosynthesis acetyltransferases) and *wzt* (a gene which encodes for an ATP-binding protein of the LPS O-antigen transport system). Genes *algE*, *algJ*, *wcaK*/*amsJ*, *epsA*, *epsG*, *sacB* and *lptC* were the least abundant, with just a few reads annotated. Multilevel model analysis revealed depth as the main factor affecting the distribution pattern of the investigated genes. Specifically, the relative abundance of *wcaF* and *lptFG* decreased with depth by half. In addition, the *epsA* gene (encoding for an EPS biosynthesis tyrosine kinase modulator) was influenced by interaction of depth and tillage. This gene was more abundant in 0–10 cm under RT, compared to 20–50 cm under CT, and no reads were detected in 10–20 cm under both CT and RT. The majority of the analyzed genes, namely *wza*, *algEJ*, *wcaB*, *wcaK/amsJ*, *kpsE*, *epsG*, *sacB*, *wzt* and *lptC*, were not significantly affected by either tillage or depth.

### Investigation of potential EPS/LPS producers

One hundred thirty-eight bacterial families harbored the investigated genes, including all dominant families (Fig. 1b). The highest numbers of sequences related to EPS and LPS synthesis and excretion (> 0.001%) were assigned to *Chitinophagaceae*, *Nitrospiraceae* and *Planctomycetaceae*. *Anaerolineaceae*, despite their high abundance, harbored a very low number of copies of the investigated genes (< 0.0002%).

The NMDS (Additional file 4B) plot once again revealed depth as the main factor affecting the distribution of the investigated genes among bacterial families. However, the influence of depth was much less pronounced than in case of the general bacterial community (Additional file 4A). This was confirmed with a multilevel model. The overall relative abundances of the investigated genes were impacted by depth in four families affiliated with EPS/LPS synthesis and excretion, while tillage had no influence, and interaction had an effect on one family only. The full list of influenced families can be taken from Additional file 5.

**Fig. 3:**
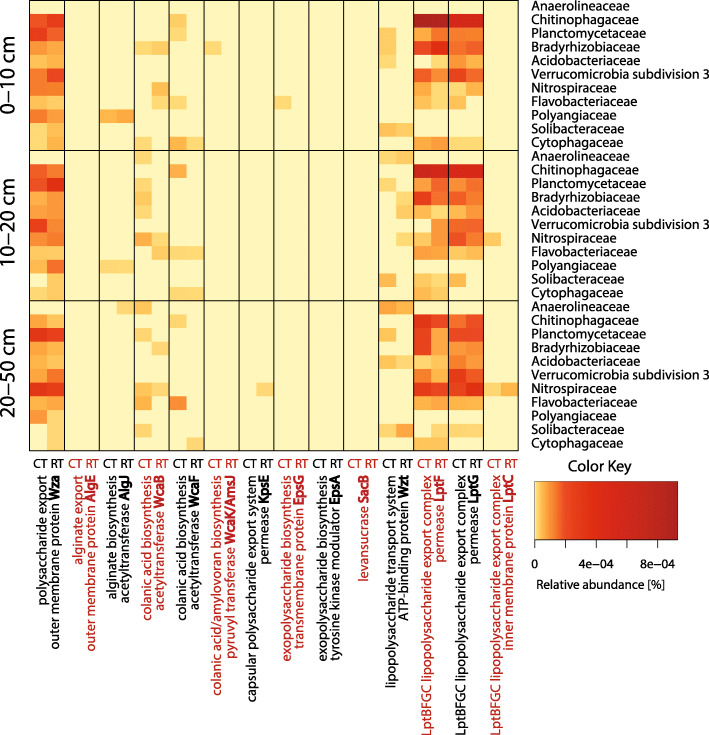
Genes encoding for proteins involved in EPS and LPS biosynthesis found in the dominant families. Heatmap representation of the mean relative numbers of genes encoding for proteins involved in EPS and LPS synthesis and excretion found in the dominant bacterial families whose abundance exceeded 0.5% in the samples from Frick taken at three depths. “CT” and “RT” on the x axis stand for “conventional tillage” and “reduced tillage”, respectively. Functional genes were assigned using hidden Markov models (HMMs) obtained from the TIGRFAMs and Pfam databases, and then sequences derived from the Kyoto Encyclopedia of Genes and Genomes (KEGG) Orthology database. Taxonomic assignment was performed against the National Center for Biotechnology Information Non-Redundant (NCBI-NR) protein sequences database

Taxonomic affiliation of the individual genes encoding for proteins involved in EPS and LPS biosynthesis was analyzed using a heatmap (Fig. 3). The most abundant genes, *wza* and *lptFG*, were harbored by most of the dominant families. *Anaerolineaceae* had neither *wza* nor *lptFG*, but harbored the *wzt* gene, which is part of the same LPS synthesis pathway. Moreover, *Polyangiaceae* carried the *wza* gene, but showed no potential to produce LPS. The other investigated genes were not so widely distributed among the dominant families. In particular, *algE*, *epsA* and *sacB*, encoding respectively for alginate export outer membrane protein, exopolysaccharide biosynthesis tyrosine kinase modulator and levansucrase, were not detected in any of the dominant families. As shown by means of a multilevel model, the gene copy numbers of *wcaF*, *epsA*, *sacB*, *wzt* and *lptC* were influenced by interaction of tillage and depth in *Chitinophagaceae*, *Bacillaceae*, *Micrococcaceae*, *Candidatus Brocadiaceae* and *Sulfuricellaceae*, whilst the abundances of *algJ* and *lptC* changed with depth in *Polyangiaceae* and *Sphingomonadaceae*, respectively.

**Fig. 4:**
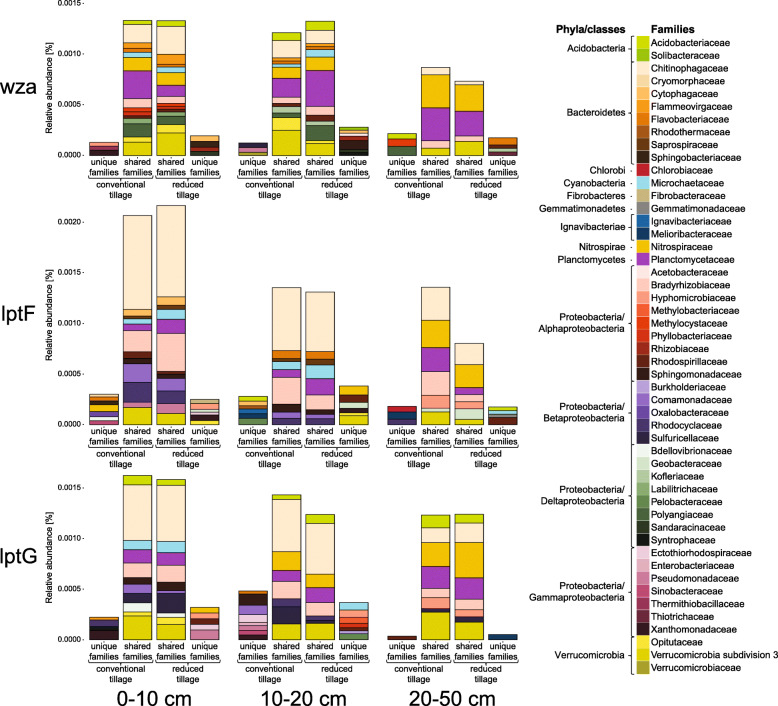
Taxonomic assignment of the *wza*, *lptF* and *lptG* genes. Mean relative abundances of the *wza*, *lptF* and *lptG* genes, encoding respectively for a polysaccharide outer membrane exporter and permeases of the LptBFGC LPS export complex, in the samples taken at three depths from plots under conventional and reduced tillage managements. Displayed are the distributions of gene copies among bacterial families listed on the right side of the graph. Only families found in at least two out of three replicates are presented. The color code is arranged according to the phylogenetic affiliation of the respective families. Compared are gene abundances in families harboring the respective genes uniquely under conventional or reduced tillage and families harboring the genes under both tillage managements. Functional genes were assigned using hidden Markov models (HMMs) obtained from the TIGRFAMs and Pfam databases, and then sequences derived from the Kyoto Encyclopedia of Genes and Genomes (KEGG) Orthology database. Taxonomic assignment was performed against the National Center for Biotechnology Information Non-Redundant (NCBI-NR) protein sequences database

Since *wza* and *lptFG* were dominating among the investigated genes, their taxonomic affiliation was analyzed in more detail. These genes were present in a total of 50 families associated with 11 phyla. The core microbiomes harboring the respective genes under both tillage treatments were identified at each sampled depth (Fig. 4). At each depth, on average ten families carried the respective genes under both tillage managements, while five were unique for either CT or RT. Overall, the three genes harbored by the core families accounted for 22.7% of all reads assigned to all the investigated genes, while 1.8 and 2.1% were unique for CT and RT, respectively. The diversity of families carrying *wza* and *lptG* significantly decreased with depth (Additional file 8). Depth triggered a decrease of *wza* and *lptFG* in *Chitinophagaceae*. The relative number of *wza* gene copies decreased with depth also in *Flammeovirgaceae* and *Labilitrichaceae*. Furthermore, depth caused a decrease of *lptG* in *Bdellovibrionaceae*, but *lptF* increased in *Nitrospiraceae*. Finally, the interaction of depth and tillage affected *lptG* in *Pseudomonadaceae*. This gene was more abundant in 0–10 cm under RT, compared to 20–50 cm under CT, and no reads were detected in the other samples.

## Discussion

### Different factors could affect the stable aggregate fraction in soil

EPS and LPS are of great importance for agricultural soils, as they reduce soil erodibility by improving soil structure [44]. However, tillage disrupts soil aggregates and alters soil physical and chemical properties. These include bulk density, pore structure, water availability and soil organic carbon [45]. Thus, changes in bacterial communities are likely to occur. This has been reported by multiple studies [46–50]. Especially CT disturbs bacterial habitats and dilutes nutrient pools by mixing topsoil with subsoil. In our study, soil organic carbon (SOC), dissolved organic carbon (DOC) and microbial biomass carbon (Cmic) had higher values in the tillage horizon under RT compared to CT. This corresponds to the data found in the literature [23, 51–53]. The increase of Cmic suggests that the absolute number of bacteria capable of synthesizing EPS and LPS should be higher under RT. Thus, we assumed that the higher DOC concentrations promotes bacteria which are able to produce EPS and LPS, and that the stable aggregate fraction (SAF) of the soil is higher under RT. Surprisingly, at our sampling site, SAF was comparable between the two tillage systems and increased significantly only below the tillage horizon. However, this might be caused by soil physical properties. Specifically, the clay content (45%) was very high at our site. Meta-analysis performed by Cooper et al. [7] suggests that the differences between tillage systems could be more pronounced in soils with a lower clay content (< 40%). Building good soil structure is more challenging in light, sandy soils, as they lack the fine particles necessary to form stable soil aggregates [54]. Conversely, soil biology has a strong influence on SAF. This includes the activity of bacteria, fungi, earthworms and plants. On one hand, the effect of plants and earthworms is rather indirect and includes for example cast formation by earthworms or increasing microbial activity by the release of organic substances to the soil via the rhizosphere of plants [15, 16]. On the other hand, bacteria and fungi directly promote aggregate formation by the excretion of gluing agents such as EPS, LPS and fungal glycoproteins, or by physical binding of soil particles by fungal mycelium [15]. Similar to the general increase of Cmic in the topsoil under RT, Kuntz et al. [55] also observed higher fungal abundances in that soil layer.

While it is obvious that ploughing physically disturbs fungal hyphae and consequently aggregates connected to them, the effect on the bacterial potential to promote aggregate formation can be much more subtle. Especially EPS composition and regulation of the respective genes is species-specific, thus a shift in the bacterial community strongly influences their potential to promote aggregate formation. To detect changes in the bacterial potential to produce EPS and LPS, we applied a metagenomic approach. As many proteins or their functional domains from genes encoding for EPS and LPS biosynthesis pathways are associated with other cellular activities as well [56], we used a pipeline combining hidden Markov model (HMM) searches with blasts against sequences derived from the Kyoto Encyclopedia of Genes and Genomes (KEGG) database to target selected genes specific for our functions of interest. Although the contribution of fungi to aggregate formation is well accepted, our analysis exclusively focused on bacteria due to the well-described biases of the existing databases towards bacteria [57]. This is also visible in our dataset, where 55.8% of sequences were assigned to bacteria, while only 1.3% could be assigned to fungi. Moreover, fungal genes require long reads for accurate annotation due to many intronic sequences [58].

### Key genes encoding for selected EPS and LPS biosynthesis pathways

Identified as key components of the analyzed EPS and LPS synthesis and excretion pathways were *wza* and *lptFG*, which encode for an outer membrane protein Wza and permeases of the LptBFGC LPS export complex (LptF and LptG), respectively. Wza acts as a translocation channel across the outer membrane for a variety of exopolysaccharides in a wide range of taxa. It is also characterized by the presence of a very well conserved polysaccharide export sequence domain (pfam 02563) [20]. Similarly, LptF and LptG are essential for transport of mature LPS to the outer membrane. These two proteins are highly conserved among Gram-negative bacteria, unlike another component of the LptBFGC transport complex, LptC [59, 60]. Benedet et al. [61] recently reported the isolation of mutants lacking LptC and suggested its supportive role in the LPS translocation. In our study, the respective gene, *lptC*, had just a few reads annotated and was detected in only one of the dominant families, *Nitrospiraceae*, even though all of them belong to phyla known to produce LPS [62]. The family *Nitrospiraceae* is essential for nitrification, and thus its high abundance in agricultural soils is expected [63].

Less abundant genes were *wcaBF* and *wzt*, encoding for the colanic acid biosynthesis acetyltransferases WcaB and WcaF, and an ATP-binding protein Wzt of the LPS O-antigen transport system, respectively. Relatively high abundances of the genes from the colanic acid biosynthesis pathway are not surprising, as colanic acid is one of the most common exopolysaccharides. However, it is also one of the exopolysaccharides secreted by Wza. Therefore, *wcaBF* were less abundant than *wza* due to their lower universality [19]. In contrast, *wzt* is involved in translocating the O-antigen to the outer leaflet of the inner membrane where it gets ligated to the other parts of LPS [64]. Thus, the lower abundance of *wzt* compared to *lptFG* can be explained by the fact that the O-antigen is not an essential component of LPS [22].

Finally, a very low number of reads was assigned to the other investigated genes, which catalyze the biosynthesis and export of alginate, colanic acid, levan and other extracellular and capsular polysaccharides. EPS biosynthesis pathways are generally poorly conserved and often species or strain-specific, so this result was expected [19, 20, 65]. Also the low abundances of *algEJ*, encoding for alginate export outer membrane protein AlgE and alginate biosynthesis acetyltransferase AlgJ, were understandable because alginate is produced by various bacteria from the genera *Pseudomonas* and *Azotobacter* [66]. These genera belong to the family *Pseudomonadaceae*, which was not dominant in our metagenomes. *Pseudomonadaceae* contains many plant growth promoting endophytes and rhizobacteria [67]. Its low abundance could be related to the poorly established vegetation at the time of sampling (March) and the fact that bulk soil samples were investigated instead of rhizosphere samples.

### EPS and LPS biosynthesis is important in agricultural soils, yet could be easily hindered

In our metagenomes, almost half (46.6%) of all bacterial families harbored genes affiliated with EPS and LPS biosynthesis. These included all dominant families whose abundance exceeded 0.5%. The ability to form EPS or LPS seems to be, therefore, an important trait for bacteria living in agricultural soils.

Despite the fact that all families dominating in our metagenomes harbored genes encoding for EPS or LPS biosynthesis, none of them accommodated genes from more than one of the investigated EPS biosynthesis pathways. This is not surprising, as few bacteria are known to produce more than one type of EPS [68]. Nonetheless, some of the analyzed genes were not represented in any of the dominant families. This could have several reasons, including: (i) that those genes might be harbored by low abundant families only, which were below the detection limit of our approach, or (ii) no genome of a representative taxon was sequenced so far. This is very likely, as databases for sequencing analysis are still biased towards fast-growing bacteria, while soil contains many slow-growing bacteria, which are difficult to isolate and culture.

### Bacterial potential to produce EPS and LPS is affected by the interplay of tillage type and tillage depth

Tillage not only disrupts soil aggregates, but also alters soil physical and chemical properties. Especially CT disturbs microbial habitats and reduces available nutrients by mixing topsoil with subsoil. Since bacterial polysaccharides contribute to soil aggregation, which was reported to be lower under CT [10, 11], and EPS and LPS production requires a lot of carbon, we hypothesized that CT weakens the bacterial potential to produce soil structure-stabilizing agents. Contradictory to this hypothesis, but similar to the SAF results in our study, the tillage system influenced bacterial community composition and the potential to synthesize EPS and LPS only in the context of the depth factor. Direct effects of tillage were visible only when closely analyzing the taxonomic affiliation of the key genes of the investigated EPS and LPS biosynthesis pathways (*wza*, *lptFG*). At each soil depth, unique families harboring the respective genes under either CT or RT were described. These findings are in accordance with the theory about functional redundancy, which states that different taxa are able to perform the same functions under changed conditions [69]. However, EPS and LPS produced by different bacteria may differ in quality, and can have different gluing properties [70, 71]. Therefore, the differences in aggregate preservation observed in other studies could be related to the differences in the properties of EPS and LPS produced under CT and RT.

The fact that the differences between tillage systems at our sampling sites were not more pronounced in comparison to other studies, is surprising, but not unprecedented. In fact, our results are in agreement with other functional analyses of agricultural soils. The work of de Vries et al. [58], who also compared CT and RT using metagenomics, and Grafe et al. [72], who compared different fertilization regimes, found little significant effects on bacterial community structure and functionality. Both studies implied that under long-term management, bacterial communities are very stable and hardly differ between treatments. In fact, it is more likely that regulation takes place on the RNA level, as tillage alters soil conditions, and thus might influence metabolic activity of soil structure-stabilizing bacteria. Ultimately, the yield of EPS and LPS could be increased or decreased by multiple factors, e.g. carbon sources or oxygen availability [18]. Thus, a metatranscriptomic analysis of the soil samples should be the next step. Ideally, omics data and SAF measurements should be correlated with the content of bacterial polysaccharides in soil. Redmile-Gordon et al. [73] made efforts to evaluate the suitability of different extracellular polymeric substances extraction methods for this medium. However, the existing methodologies are still biased and do not allow for distinction between polysaccharides of different origins (bacterial, fungal, plant, etc.). Therefore, further research needs to address these issues in order to establish a standardized protocol.

### RT and CT promote the potential to produce EPS and LPS in bacteria

To our knowledge, previous metagenomic comparisons of tillage systems encompassed only surface soil samples [46–50]. However, other studies on tillage included analyses of chemical and physical properties of soil also at deeper levels [74–76]. The studies revealed that tillage can differentially impact bacterial habitats of different soil layers. Gadermaier et al. [4] demonstrated, that also at our sampling site the effects of tillage on soil organic carbon, microbial biomass and soil nutrients, varied with the depth of sampling. Therefore, we expected that bacterial communities at different depths would be differently affected by tillage, prompting the inclusion of a depth factor in our metagenomic analysis.

It is well-known that bacterial communities change with depth in undisturbed soils [77–79]. We showed that the composition of bacterial families in the upper soil depths (in the tillage horizon) differs from the one in lower soil depths (below the tillage horizon) also in tilled soils. This happens because specific conditions of different soil depths select for the best-adapted microorganisms. That is to say, deeper soil layers are generally more oxygen-depleted and nutrient-poor than upper soil layers. In the deepest soil layer which we sampled, the dominant family was *Anaerolineaceae*. Unsurprisingly, its members are strictly anaerobic oligotrophs [80, 81]. Including *Anaerolineaceae*, 34.8% of bacterial families detected in our metagenomes were significantly influenced by depth. Furthermore, we showed that in tilled soils, depth has not only a big influence on bacterial community composition, but also on relative abundances of genes involved in EPS and LPS synthesis and excretion. The relative abundances of *wza*, *wcaF* and *lptFG* were higher in the upper soil layers. Additionally, *epsA* was influenced by the interaction of tillage and depth, but its low abundance undermines the significance of this finding. Moreover, the diversity of bacterial families which harbored *wza* and *lptG*, two out of three most abundant genes of the analyzed biosynthesis pathways, decreased with depth. These effects should be even more pronounced due to the stratification of Cmic, which was significantly higher under both CT and RT, in the 0–20 cm layers as opposed to the 20–50 cm layers. Although we expected higher potential to produce EPS and LPS in the deeper, undisturbed soil layers, these observations suggest that EPS and LPS synthesis plays a bigger role in the surface soil layers, which are regularly disturbed by tillage. This could be explained by better aeration and availability of nutrients in the tillage horizon, as these parameters are known to be important for EPS and LPS production [18, 82, 83]. Otherwise, Galant et al. [84] postulated that disturbances increase the diversity and productivity of bacteria performing important ecological functions, which also coincides with our results. In our study, the disturbance caused by tillage could select for bacteria which are capable of synthesizing protective compounds, such as EPS and LPS.

Finally, it is difficult to separate depth and tillage effects, as the depth effects might be also induced by tillage. The stratification of soil chemical and physical properties in our study was artificially induced by tillage [4]. In particular, soil organic carbon (SOC) steadily decreased with depth under both CT and RT. By introducing such changes in soil properties along the soil profile, tillage indirectly caused the shifts in bacterial communities allocated as the effects of depth. Those shifts could be driven primarily by the disturbance caused by tillage. Specifically, tillage could stir the established bacterial communities in the tillage horizon, making it possible for new taxa to emerge. At the same time, a long-term competition in the undisturbed soil layers below the tillage horizon would enable only the best-adapted bacteria to thrive. This type of competition-driven dominance of selected taxa is well-known in ecological communities [85, 86]. Moreover, it has recently been demonstrated that periodic disturbances have an impact on bacterial communities by promoting the cohabitation of ecologically different bacteria [84]. In conclusion, as similar effects of depth were detected under both CT and RT, the impact of tillage in general, might be more selective than the subtle differences between these two systems.

## Conclusions

Although a typical stratification of soil carbon and microbial biomass was observed under RT in our study, no difference in the stable aggregate fraction of the soil or the potential to produce EPS and LPS was observed between RT and CT systems. While the potential to produce EPS and LPS was enhanced in the tillage horizon, tillage affected the taxonomic affiliation of genes encoding for proteins involved in the biosynthesis of specific EPS and LPS. These compounds can have different properties depending on the bacterial producers. Thus, the regulation of EPS and LPS formation can take place at two levels: (i) even small changes in the bacterial community composition could disturb the overall capacity of EPS and LPS to stabilize soil structure, or (ii) regulation takes place on the level of gene expression. Consequently, future studies need to figure out under which conditions the potential to produce EPS and LPS is recalled. However, the fast turnover of mRNA would require another sampling strategy which accounts for that dynamic, such as high resolved samplings throughout the season and the day, as beside tillage, also carbon input by plants and fertilization might influence expression of the respective genes. Moreover, soil at the sampling site was already well-structured due to its high clay content. We expect a stronger effect of tillage in sandy soils, which lack the fine particles necessary to form stable soil aggregates.

## Additional file

Additional file 1: Agricultural practices applied in the experimental field in Frick prior to sampling. (DOC 28 kb)

Additional file 2: KO numbers related to EPS or LPS production found in the online Kyoto Encyclopedia of Genes and Genomes (KEGG) Orthology database (October 2016). (DOC 26 kb)

Additional file 3: Details of the sequencing run. Shown are the numbers of obtained reads, total length of the reads and average read length per sample before and after quality filtering. “C” and “R” at the beginning of sample names stand for either “conventional tillage” or “reduced tillage”, respectively. The following “A”, “B” and “C” stand for the sampling depth (A – 0-10 cm, B – 10-20 cm and C – 20-50 cm). (DOC 43 kb)

Additional file 4: NMDS ordination plots depicting taxonomic profiles of bacteria at the family level in conventional and reduced tillage-treated soils sampled at three different depths. Ellipses drawn around triplicates represent a 95% confidence level. Shown is A) overall community, and B) affiliation of genes related to EPS and LPS synthesis. Each point in the plot represents a different sample (*n* = 18), and the location of the points is based on Bray-Curtis distances. Taxonomic assignment was performed against the National Center for Biotechnology Information Non-Redundant (NCBI-NR) protein sequences database. Functional genes were assigned using hidden Markov models (HMMs) obtained from the TIGRFAMs and Pfam databases, and then sequences derived from the Kyoto Encyclopedia of Genes and Genomes (KEGG) Orthology database. (PDF 152 kb)

Additional file 5: Bacterial families whose relative abundances and potential to produce EPS or LPS were significantly affected depth or interaction of tillage and depth. Significant differences between the treatments were determined by a multilevel model (*n* = 3, *p* < 0.05). (DOC 30 kb)

Additional file 6: Comparison of the 35 most abundant bacterial families according to taxonomic annotations based on the NCBI-NR and SILVA databases. (PDF 248 kb)

Additional file 7: Rarefaction curves of metagenomic datasets derived from conventional and reduced tillage-treated soils sampled at three different depths. Depicted is the number of assigned genes involved in EPS and LPS production as a function of sequencing depth. The genes were assigned using hidden Markov models (HMMs) obtained from the TIGRFAMs and Pfam databases, and then sequences derived from the Kyoto Encyclopedia of Genes and Genomes (KEGG) Orthology database. “C” and “R” at the beginning of sample names stand for either “conventional tillage” or “reduced tillage”, respectively. The following “A”, “B” and “C” stand for the sampling depth (A – 0-10 cm, B – 10-20 cm and C – 20-50 cm). (JPEG 1320 kb)

Additional file 8: Boxplot depicting Shannon-Weiner index values which describe the diversity of bacterial families harboring genes *wza*, *lptF* and *lptG* at three depths. Significant influence of depth, but not tillage, was detected when applying a multilevel model analysis (n = 3). Therefore, tillage treatments were pooled for this plot. The influence of depth is symbolized with “*”. Significance levels are represented by the amount of symbols: 1 – p < 0.05, 2 – *p* < 0.01, 3 – *p* < 0.001. (PDF 117 kb)

## Abbreviations

Cmic: Microbial biomass carbon
CT: Conventional tillage
DOC: Dissolved organic carbon
EPS: Exopolysaccharides
LPS: Lipopolysaccharides
RT: Reduced tillage
SAF: Stable aggregate fraction
SOC: Soil organic carbon
