# Supplementary material for: A long-term field experiment demonstrates the influence of tillage on the bacterial potential to produce soil structure-stabilizing agents such as exopolysaccharides and lipopolysaccharides
Source: Environ Microbiome. 2019 Mar 28;14:1. doi: 10.1186/s40793-019-0341-7 (PMC7989815; doi:10.1186/s40793-019-0341-7)
Supplement: Supplementary file 1 — Agricultural practices applied in the experimental field in Frick prior to sampling. (DOC 28 kb) [file 40793_2019_341_MOESM1_ESM.doc]

| **Date** | **Procedure** |
| --- | --- |
| **20.10.2013** | Winter wheat seeding |
| **19.03.2014** | Slurry application, 69 kg total N/ha |
| **09.04.2014** | Slurry application, 57 kg total N/ha |
| **17.07.2014** | Winter wheat harvest |
| **25.08.2014** | Green manure seeding |
| **17./18.03.2015** | Soil sampling |
