# Supplementary material for: A long-term field experiment demonstrates the influence of tillage on the bacterial potential to produce soil structure-stabilizing agents such as exopolysaccharides and lipopolysaccharides
Source: Environ Microbiome. 2019 Mar 28;14:1. doi: 10.1186/s40793-019-0341-7 (PMC7989815; doi:10.1186/s40793-019-0341-7)
Supplement: Supplementary file 2 — KO numbers related to EPS or LPS production found in the online Kyoto Encyclopedia of Genes and Genomes (KEGG) Orthology database (October 2016). (DOC 26 kb) [file 40793_2019_341_MOESM2_ESM.doc]

| **EPS (67)** | K00689, K00692, K00694, K00752, K00903, K01991, K03207, K03208, K03606, K03818, K03819, K09688, K09689, K10107, K13620, K13650, K13654, K13683, K13684, K16081, K16552, K16553, K16554, K16555, K16556, K16557, K16558, K16560, K16561, K16562, K16563, K16564, K16565, K16566, K16567, K16568, K16692, K16696, K16700, K16701, K16702, K16703, K16708, K16709, K16710, K16711, K16712, K16713, K19292, K19293, K19294, K19295, K19296, K19418, K19419, K19420, K19421, K19422, K19424, K19425, K19426, K19427, K19428, K19429, K19430, K19431, K19667 |
| --- | --- |
| **LPS (14)** | K05399, K05790, K06861, K07091, K07271, K08280, K08992, K09690, K09691, K09774, K11719, K11720, K16695, K19804 |
