# Supplementary material for: A long-term field experiment demonstrates the influence of tillage on the bacterial potential to produce soil structure-stabilizing agents such as exopolysaccharides and lipopolysaccharides
Source: Environ Microbiome. 2019 Mar 28;14:1. doi: 10.1186/s40793-019-0341-7 (PMC7989815; doi:10.1186/s40793-019-0341-7)
Supplement: Supplementary file 3 — Details of the sequencing run. Shown are the numbers of obtained reads, total length of the reads and average read length per sample before and after quality filtering. “C” and “R” at the beginning of sample names stand for either “conventional tillage” or “reduced tillage”, respectively. The following “A”, “B” and “C” stand for the sampling depth (A – 0-10 cm, B – 10-20 cm and C – 20-50 cm). (DOC 43 kb) [file 40793_2019_341_MOESM3_ESM.doc]

| **Raw data** | **CA1** | **CA2** | **CA3** | **CB1** | **CB2** | **CB3** | **CC1** | **CC2** | **CC3** |
| --- | --- | --- | --- | --- | --- | --- | --- | --- | --- |
| **Number of reads** | 2549636 | 2708128 | 2387144 | 1956536 | 2095558 | 2372948 | 1675552 | 1761742 | 2084406 |
| **Total length of reads** | 767440436 | 815146528 | 718530344 | 588917336 | 630762958 | 714257348 | 504341152 | 530284342 | 627406206 |
| **Average length of reads** | 301 | 301 | 301 | 301 | 301 | 301 | 301 | 301 | 301 |
| **Filtered data** |  |  |  |  |  |  |  |  |  |
| **Number of reads** | 2549495 | 2707738 | 2387077 | 1956447 | 2095401 | 2372859 | 1675291 | 1761533 | 2083272 |
| **Total length of reads** | 757415945 | 805059148 | 709958547 | 581913254 | 623067219 | 705306628 | 497711661 | 522694430 | 611096918 |
| **Average length of reads** | 297.08 | 297.32 | 297.42 | 297.43 | 297.35 | 297.24 | 297.09 | 296.73 | 293.34 |
|  |  |  |  |  |  |  |  |  |  |
| **Raw data** | **RA1** | **RA2** | **RA3** | **RB1** | **RB2** | **RB3** | **RC1** | **RC2** | **RC3** |
| **Number of reads** | 2355348 | 2178762 | 2670446 | 2030052 | 2318146 | 2442574 | 1659310 | 2252744 | 1812660 |
| **Total length of reads** | 708959748 | 655807362 | 803804246 | 611045652 | 697761946 | 735214774 | 499452310 | 678075944 | 545610660 |
| **Average length of reads** | 301 | 301 | 301 | 301 | 301 | 301 | 301 | 301 | 301 |
| **Filtered data** |  |  |  |  |  |  |  |  |  |
| **Number of reads** | 2355296 | 2178624 | 2670386 | 2030004 | 2318025 | 2442433 | 1659110 | 2252382 | 1812502 |
| **Total length of reads** | 700755406 | 648213723 | 792113860 | 603953567 | 689473640 | 726293001 | 493120371 | 668547107 | 538679590 |
| **Average length of reads** | 297.52 | 297.53 | 296.63 | 297.51 | 297.44 | 297.36 | 297.22 | 296.82 | 297.20 |
