# Supplementary material for: A long-term field experiment demonstrates the influence of tillage on the bacterial potential to produce soil structure-stabilizing agents such as exopolysaccharides and lipopolysaccharides
Source: Environ Microbiome. 2019 Mar 28;14:1. doi: 10.1186/s40793-019-0341-7 (PMC7989815; doi:10.1186/s40793-019-0341-7)
Supplement: Supplementary file 4 — NMDS ordination plots depicting taxonomic profiles of bacteria at the family level in conventional and reduced tillage-treated soils sampled at three different depths. Ellipses drawn around triplicates represent a 95% confidence level. Shown is A) overall community, and B) affiliation of genes related to EPS and LPS synthesis. Each point in the plot represents a different sample (n = 18), and the location of the points is based on Bray-Curtis distances. Taxonomic assignment was performed against the National Center for Biotechnology Information Non-Redundant (NCBI-NR) protein sequences database. Functional genes were assigned using hidden Markov models (HMMs) obtained from the TIGRFAMs and Pfam databases, and then sequences derived from the Kyoto Encyclopedia of Genes and Genomes (KEGG) Orthology database. (PDF 152 kb) [file 40793_2019_341_MOESM4_ESM.pdf]

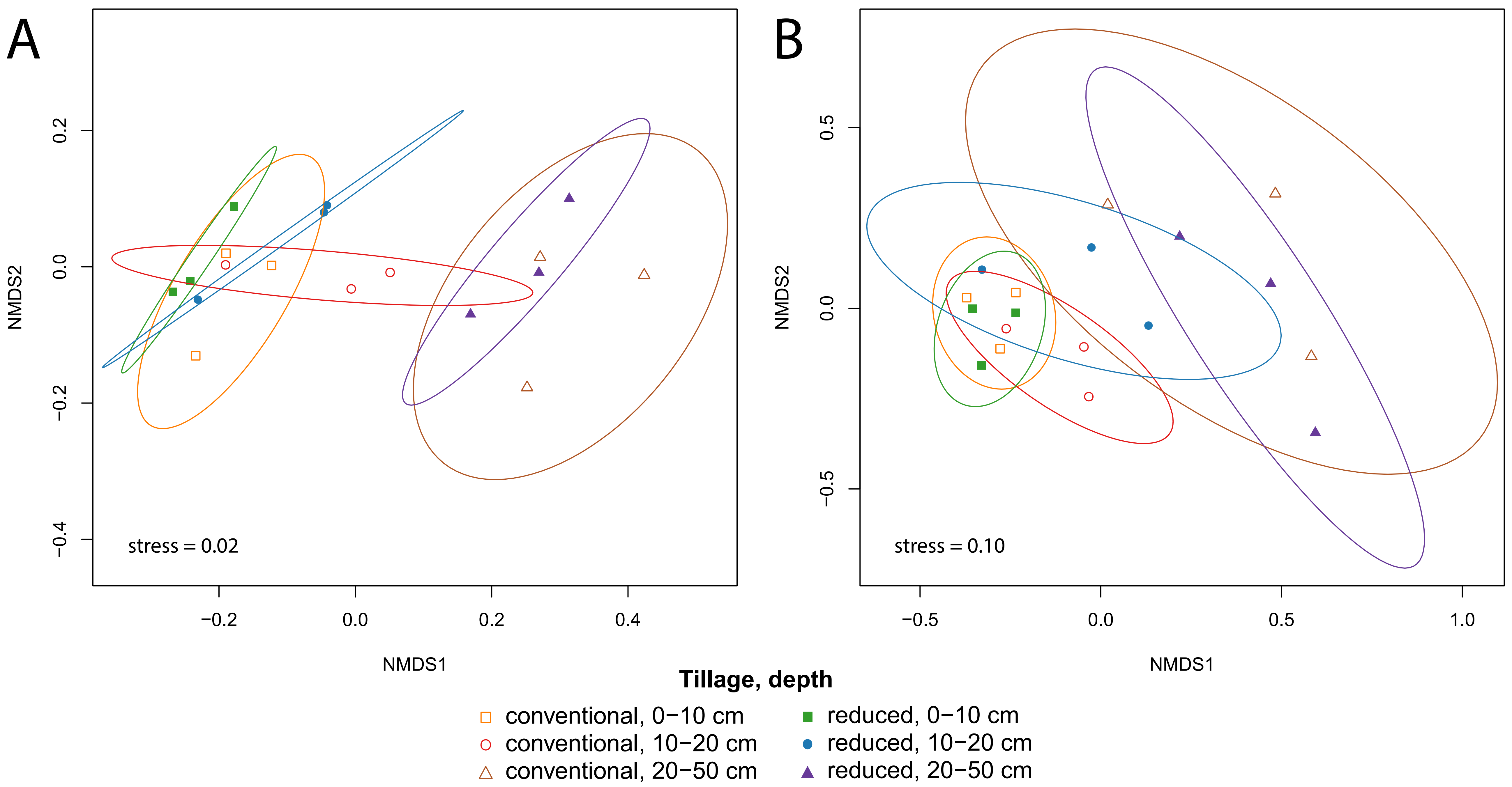
A
B
NMDS2
NMDS1
stress = 0.02
stress = 0.10
0.2
0.0
−0.2
−0.4
0.4
0.5
−0.5
1.0
Tillage, depth
conventional, 0−10 cm
conventional, 10−20 cm
conventional, 20−50 cm
reduced, 0−10 cm
reduced, 10−20 cm
reduced, 20−50 cm
