# Supplementary material for: A long-term field experiment demonstrates the influence of tillage on the bacterial potential to produce soil structure-stabilizing agents such as exopolysaccharides and lipopolysaccharides
Source: Environ Microbiome. 2019 Mar 28;14:1. doi: 10.1186/s40793-019-0341-7 (PMC7989815; doi:10.1186/s40793-019-0341-7)
Supplement: Supplementary file 5 — Bacterial families whose relative abundances and potential to produce EPS or LPS were significantly affected depth or interaction of tillage and depth. Significant differences between the treatments were determined by a multilevel model (n = 3, p < 0.05). (DOC 30 kb) [file 40793_2019_341_MOESM5_ESM.doc]

| **Factor** | **Families whose abundance was affected** | **Families whose potential to produce EPS/LPS was affected** |
| --- | --- | --- |
| **Depth** | *Acetobacteraceae, Acidimicrobiaceae, Alcaligenaceae, Alicyclobacillaceae, Anaerolineaceae, Aquificaceae, Ardenticatenaceae, Bacillaceae, Bdellovibrionaceae, Bogoriellaceae, Bradyrhizobiaceae, Burkholderiaceae, Caldilineaceae, Caldisericaceae, Candidatus Actinomarinaceae, Candidatus.Brocadiaceae, Carnobacteriaceae, Caulobacteraceae, Cellulomonadaceae, Chitinophagaceae, Chlorobiaceae, Chloroflexaceae, Chromobacteriaceae, Comamonadaceae, Conexibacteraceae, Corynebacteriaceae, Cyclobacteriaceae, Cytophagaceae, Dehalococcoidaceae, Deinococcaceae, Desulfobacteraceae, Desulfohalobiaceae, Ectothiorhodospiraceae, Erythrobacteraceae, Flammeovirgaceae, Geodermatophilaceae, Gordoniaceae, Herpetosiphonaceae, Hydrogenophilaceae, Hyphomicrobiaceae, Hyphomonadaceae, Ignavibacteriaceae, Intrasporangiaceae, Kofleriaceae, Ktedonobacteraceae, Labilitrichaceae, Magnetococcaceae, Marinilabiliaceae, Melioribacteraceae, Methylobacteriaceae, Microbacteriaceae, Microchaetaceae, Micrococcaceae, Mycobacteriaceae, Nakamurellaceae, Nannocystaceae, Nitrosomonadaceae, Nitrospiraceae, Nocardioidaceae, Nostocaceae, Opitutaceae, Oscillochloridaceae, Oxalobacteraceae, Paenibacillaceae, Patulibacteraceae, Peptococcaceae, Phycisphaeraceae, Phyllobacteriaceae, Polyangiaceae, Porphyromonadaceae, Propionibacteriaceae, Rhodobacteraceae, Rhodobiaceae, Rhodospirillaceae, Rivulariaceae, Roseiflexaceae, Rubrobacteraceae, Sandaracinaceae, Saprospiraceae, Schleiferiaceae, Scytonemataceae, Sinobacteraceae, Solirubrobacteraceae, Sphingobacteriaceae, Sphingomonadaceae, Spirochaetaceae, Sulfuricellaceae, Sutterellaceae, Syntrophaceae, Syntrophobacteraceae, Syntrophomonadaceae, Syntrophorhabdaceae, Thermaceae, Thermoanaerobacteraceae, Thermogemmatisporaceae, Thiotrichaceae, Trueperaceae, Verrucomicrobiaceae, Vibrionaceae, Vulgatibacteraceae, Waddliaceae, Xanthobacteraceae, Xanthomonadaceae* | *Chitinophagaceae, Flammeovirgaceae, Labilitrichaceae, Nitrospiraceae* |
| **Interaction of tillage and depth** | *Jonesiaceae* | *Sulfuricellaceae* |
