# Supplementary material for: A long-term field experiment demonstrates the influence of tillage on the bacterial potential to produce soil structure-stabilizing agents such as exopolysaccharides and lipopolysaccharides
Source: Environ Microbiome. 2019 Mar 28;14:1. doi: 10.1186/s40793-019-0341-7 (PMC7989815; doi:10.1186/s40793-019-0341-7)
Supplement: Supplementary file 6 — Comparison of the 35 most abundant bacterial families according to taxonomic annotations based on the NCBI-NR and SILVA databases. (PDF 248 kb) [file 40793_2019_341_MOESM6_ESM.pdf]

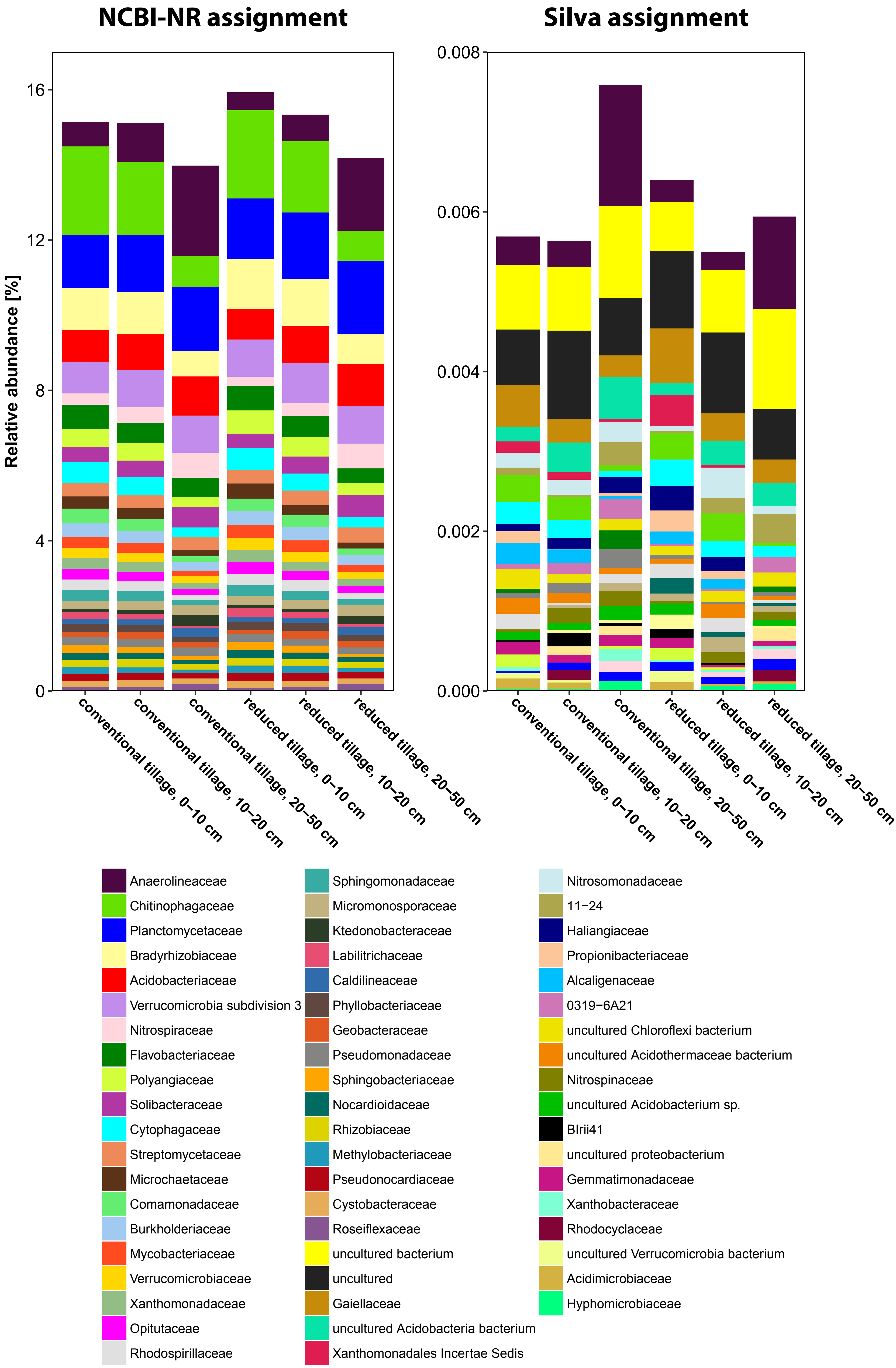

NCBI-NR assignment
Silva assignment
Relative abundance [%]
16
12
8
4
0
0.008
0.006
0.004
0.002
0.000
conventional tillage, 0–10 cm
conventional tillage, 10–20 cm
conventional tillage, 20–50 cm
reduced tillage, 0–10 cm
reduced tillage, 10–20 cm
reduced tillage, 20–50 cm
Anaerolineaceae
Chitinophagaceae
Planctomycetaceae
Bradyrhizobiaceae
Acidobacteriaceae
Verrucomicrobia subdivision 3
Nitrospiraceae
Flavobacteriaceae
Polyangiaceae
Solibacteraceae
Cytophagaceae
Streptomycetaceae
Microchaetaceae
Comamonadaceae
Burkholderiaceae
Mycobacteriaceae
Verrucomicrobiaceae
Xanthomonadaceae
Opitutaceae
Rhodospirillaceae
Sphingomonadaceae
Micromonosporaceae
Ktedonobacteraceae
Labilitrichaceae
Caldilineaceae
Phyllobacteriaceae
Geobacteraceae
Pseudomonadaceae
Sphingobacteriaceae
Nocardioidaceae
Rhizobiaceae
Methylobacteriaceae
Pseudonocardiaceae
Cystobacteraceae
Roseiflexaceae
uncultured bacterium
uncultured
Gaiellaceae
uncultured Acidobacteria bacterium
Xanthomonadales Incertae Sedis
Nitrosomonadaceae
11−24
Haliangiaceae
Propionibacteriaceae
Alcaligenaceae
0319−6A21
uncultured Chloroflexi bacterium
uncultured Acidothermaceae bacterium
Nitrospinaceae
uncultured Acidobacterium sp.
BIrii41
uncultured proteobacterium
Gemmatimonadaceae
Xanthobacteraceae
Rhodocyclaceae
uncultured Verrucomicrobia bacterium
Acidimicrobiaceae
Hyphomicrobiaceae
