# Supplementary figures and images for: A long-term field experiment demonstrates the influence of tillage on the bacterial potential to produce soil structure-stabilizing agents such as exopolysaccharides and lipopolysaccharides
Source: Environ Microbiome. 2019 Mar 28;14:1. doi: 10.1186/s40793-019-0341-7 (PMC7989815; doi:10.1186/s40793-019-0341-7)

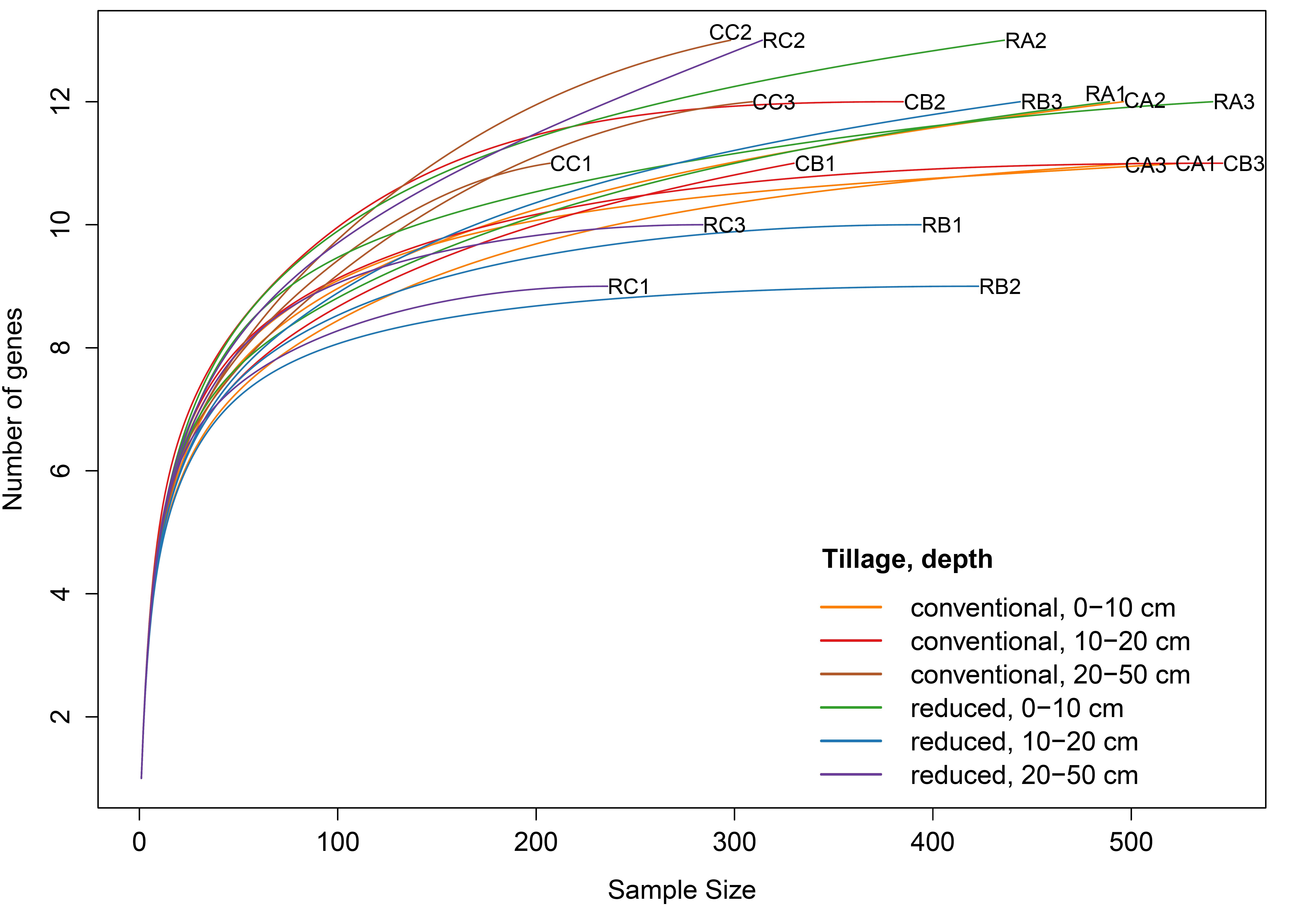

Supplement: Supplementary file 7 — Rarefaction curves of metagenomic datasets derived from conventional and reduced tillage-treated soils sampled at three different depths. Depicted is the number of assigned genes involved in EPS and LPS production as a function of sequencing depth. The genes were assigned using hidden Markov models (HMMs) obtained from the TIGRFAMs and Pfam databases, and then sequences derived from the Kyoto Encyclopedia of Genes and Genomes (KEGG) Orthology database. “C” and “R” at the beginning of sample names stand for either “conventional tillage” or “reduced tillage”, respectively. The following “A”, “B” and “C” stand for the sampling depth (A – 0-10 cm, B – 10-20 cm and C – 20-50 cm). (JPEG 1320 kb) [file 40793_2019_341_MOESM7_ESM.jpg]

Shannon-Weiner index

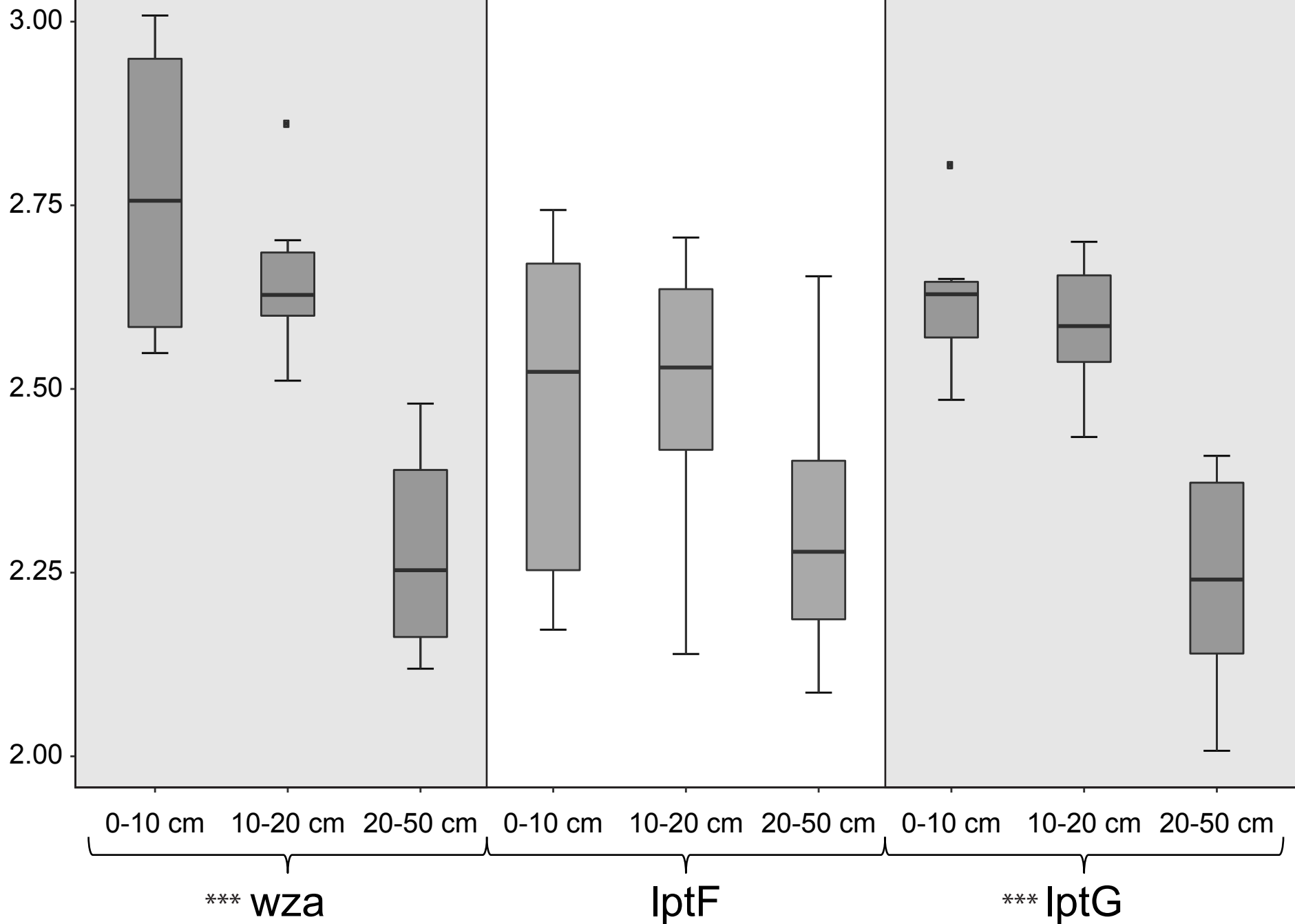

Supplement: Supplementary file 8 — Boxplot depicting Shannon-Weiner index values which describe the diversity of bacterial families harboring genes wza, lptF and lptG at three depths. Significant influence of depth, but not tillage, was detected when applying a multilevel model analysis (n = 3). Therefore, tillage treatments were pooled for this plot. The influence of depth is symbolized with “*”. Significance levels are represented by the amount of symbols: 1 – p < 0.05, 2 – p < 0.01, 3 – p < 0.001. (PDF 117 kb) [file 40793_2019_341_MOESM8_ESM.pdf]
